# Longitudinal Multimodal Monitoring of Eight Captive Beluga Whale (*Delphinapterus leucas*) Pregnancies over a 25-Year Period

**DOI:** 10.3390/ani16132062

**Published:** 2026-07-03

**Authors:** Takashi Kamio, Wataru Ohtomo, Yuichiro Akune, Masanori Kurita, Yasuo Inoshima

**Affiliations:** 1 Port of Nagoya Public Aquarium, Nagoya Port Foundation, 1-3 Minato-machi, Minato-ku, Nagoya 455-0033, Japan; 2 Laboratory of Food and Environmental Hygiene, Joint Department of Veterinary Medicine, Gifu University, 1-1 Yanagido, Gifu 501-1193, Japan; 3 Joint Graduate School of Veterinary Sciences, Gifu University, 1-1 Yanagido, Gifu 501-1193, Japan

**Keywords:** beluga whale, *Delphinapterus leucas*, parturition, pregnancy, serum progesterone concentrations, ultrasonography

## Abstract

The accurate prediction of parturition in managed beluga whales (*Delphinapterus leucas*) is fundamental for optimizing maternal and neonatal care; however, reliable predictive indicators remain limited. Here, eight pregnancies (five live births and three adverse pregnancy outcomes) monitored over 25 years at a single facility were retrospectively analyzed. The rectal temperatures, serum progesterone concentrations, gestation lengths, food intake, behavioral changes, and fetal heart rates of the whales were evaluated, particularly prepartum. Five successful pregnancies exhibited consistent seasonal timing and reproducible endocrine and physiological trajectories. The mean gestation length was 466 ± 8.4 days. The rectal temperatures of dams that delivered live offspring decreased by 1.6 ± 0.5 °C approximately 1.3 ± 0.5 days before parturition. In successful pregnancies, serum progesterone concentrations declined prepartum but typically remained detectable until parturition. In contrast, a concentration of approximately 1 ng/mL prior to parturition was observed in the pregnancy that resulted in stillbirth. Adverse pregnancy outcomes were associated with deviations from the patterns observed in successful pregnancies, including abnormal gestation length, notably reduced progesterone concentrations, altered fetal heart rate trajectories, and ultrasonographic evidence of fetal cranial asymmetry. These findings highlight the importance of integrated multimodal monitoring in predicting parturition and identifying abnormal pregnancy progression in managed beluga whales.

## Simple Summary

Predicting parturition in captive whales is essential for ensuring the safety of both the dam and calf. In beluga whales (*Delphinapterus leucas*), signs indicative of impending parturition have been previously reported. However, methods that reliably predict parturition or detect all pregnancy complications are lacking. Here, we retrospectively evaluated eight beluga whale pregnancies monitored over 25 years at a single facility. We assessed the rectal temperature, serum progesterone concentration, food intake, behavior, and fetal heart rate. In successful pregnancies, these indicators followed consistent patterns, including stable progesterone concentrations that remained detectable until shortly before birth. By contrast, pregnancies with adverse outcomes exhibited atypical findings, including abnormal gestation lengths, reduced progesterone concentrations, abnormal fetal heart rates, and fetal cranial asymmetry. Complications appeared to be associated with changes across numerous monitored indicators rather than abnormalities in a single parameter. These results suggest that combining hormonal, physical, and ultrasonographic monitoring may facilitate the prediction of parturition and identify warning indicators early in pregnancy. This multimodal approach may support reproductive management and calf survival in beluga whales under human care.

## 1. Introduction

The accurate prediction of parturition in captive cetaceans is a critical component of reproductive management [1,2,3,4,5,6]. Parturition is a critical physiological process; thus, preparation for emergency situations is important for the dam and newborn [2]. A structured protocol covering the preparation, prepartum, intrapartum, and postpartum periods has been proposed for bottlenose dolphin (*Tursiops truncatus*) reproduction, underscoring the continuous monitoring and management of dams throughout pregnancy and parturition [2]. Newborns are particularly susceptible to mortality associated with trauma, impaired postnatal growth and development, and infectious diseases, making this period the most critical for effective management of bottlenose dolphins under human care [7]. Advances in cetacean medical care have improved neonatal management in both bottlenose dolphins and beluga whales (*Delphinapterus leucas*), highlighting the importance of accurate periparturient monitoring and timely intervention [8,9].

Across cetacean species, various physiological, endocrine, ultrasonographic, and behavioral changes have been reported as indicators of impending parturition [1,2,3,4,5,6,10,11,12]. In a killer whale (*Orcinus orca*), maternal body temperature decreased by approximately 0.4 °C 2 days before parturition and by 0.7 °C 1 day before parturition [10]. Comparable declines within 12–24 h before parturition have been observed in bottlenose dolphins [2,11]. Serum progesterone concentration measurements are widely used to estimate conception dates and monitor gestation [6,12]. Ultrasonographic fetal measurements, including biparietal and thoracic diameters, enable the prediction of parturition within approximately 7 days [1,2,4,5]. Additionally, changes in fetal position immediately before parturition have also been described [1,4,5]. Food intake may decrease before birth, although this behavior varies among individuals [2,3,6]. Prepartum behavioral changes are also indicators of impending parturition [12]. Beluga whales originating from the Hudson Bay have been the focus of several reproductive studies [12,13,14], with a reported gestation length of 467 ± 5.4 days [14].

Although these studies have provided valuable reference data, the prediction of parturition based on a single parameter remains suboptimal, and the clinical interpretation of deviations from expected trajectories has not been comprehensively characterized. Additionally, improving the accuracy of parturition prediction may facilitate the early identification of abnormal fetal growth trajectories, including possible intrauterine growth restriction, thereby enhancing the clinical and conservation relevance of reproductive monitoring in this species [14]. Endocrine monitoring combined with ultrasonography has been extensively applied to determine ovulation timing and advance the development of artificial insemination techniques in odontocetes, including beluga whales [15,16,17,18].

For example, belugas from the eastern Beaufort Sea population have been reported to attain greater adult body size than those from lower-latitude populations [19]. However, because most published reproductive data in beluga whales are derived from non-Russian-origin populations, the applicability of those findings to Russian-origin belugas remains uncertain. In addition, longitudinal evaluations within a single managed population may facilitate the characterization of normal reference trajectories against which abnormal patterns can be identified. To the best of our knowledge, no comprehensive longitudinal data on pregnancy and parturition in Russian-origin belugas have been reported.

This study aimed to characterize longitudinal multimodal monitoring trajectories associated with normal and abnormal pregnancy outcomes in managed beluga whales, particularly during the prepartum period. Rectal temperature, serum progesterone concentrations, gestation length, lactation status, behavioral changes, food intake, and fetal heart rate were retrospectively evaluated in eight pregnancies (five live births and three adverse pregnancy outcomes) monitored over 25 years at the Port of Nagoya Public Aquarium (PNPA, Nagoya, Japan). As all pregnancies were monitored within a single managed population, the relative contributions of population origin and environmental or husbandry factors could not be determined. We hypothesized that deviations from expected multimodal monitoring trajectories would be associated with adverse pregnancy outcomes.

## 2. Materials and Methods

### 2.1. Animals and Sample Collection

In this retrospective observational study, we evaluated eight pregnancies that were monitored over 25 years at the Port of Nagoya Public Aquarium (PNPA, Nagoya, Japan). Owing to the limited availability of reproductive datasets in managed beluga whales, all pregnancies available during the study period were included in the analysis.

Five beluga whales experienced eight pregnancies during the 25-year study period at PNPA (Table 1). Four of the five studied females were originally wild-caught from Russia in the 1990s, and one was captive-born to parents from Russia. Throughout the study period, 14 individual beluga whales were housed at PNPA, including seven wild-caught individuals and seven individuals born at PNPA. Breeding management was conducted to minimize the risk of inbreeding. Animals housed together during the breeding season were selected based on pedigree information, where feasible. No hormonal contraceptives were used during the study period. Females were housed in an approximately 2078 m^3^ manufactured saltwater enclosure (temperature controlled at 13–18 °C). All blood samples were obtained during voluntary presentation or routine venipuncture as part of medical or management procedures from five female beluga whales at various intervals from 2001 to 2025. Conception dates were determined based on direct visual observation of copulatory behavior, supported by sustained elevation of serum progesterone concentrations and, when available, ultrasonographic detection of ovulation (SSD-900 (Aloka, Tokyo, Japan) with convex probe UST-979 (Aloka, Tokyo, Japan); Noblus (Hitachi, Tokyo, Japan) with convex probe C25 (Hitachi, Tokyo, Japan); and Arietta Prologue (Fujifilm, Tokyo, Japan) with convex probe C251 (Fujifilm, Tokyo, Japan)), as described subsequently. Further, pregnancy was confirmed by ultrasonographic visualization of the fetus. When numerous copulatory events were observed within the same reproductive cycle, the conception date was assigned to the copulatory event most consistent with the progesterone and ultrasonographic findings.

The gestational period was divided into three trimesters, which were further classified into early and late phases for evaluation (first: Days 0–78 and 79–156; second: Days 157–235 and 236–313; and third: Days 314–397 and Day 398 to parturition). The boundaries were defined based on previously reported gestational developmental stages [14]. Within the late third trimester, the period from 10 days before parturition to parturition was evaluated separately.

Data were not available for all the females in each set of observations. For periparturient food intake, values (kg/day) obtained from each animal’s record (recorded as a standard husbandry practice) were used. For trend analysis, the mean food intake was determined for all animals 15 days before and after parturition. The arching and crouching behaviors, milk leakage, and vaginal discharge of the dams were observed and recorded. Behavioral observations were conducted daily by trained husbandry staff during routine husbandry and monitoring from an underwater viewing area of the facility, where continuous observation of the whales was possible through an acrylic viewing window. Because animal care staff changed over the 25-year study period, the same observers were not involved in all pregnancies. Behavioral observations were conducted using shared practical criteria, and routine handover among staff was performed to minimize inter-observer differences as much as possible; however, formal assessment of inter-observer agreement was not performed. Arching was defined as a transient upward curvature of the body, whereas crouching was defined as a sustained ventral flexion of the body with the abdomen positioned to the pool floor closer than that during normal swimming or resting behavior. Arching and crouching behaviors were recorded when observed, and their frequency was quantified as the number of occurrences per hour. The date and time of the following events were recorded for each birth: first vaginal discharge, appearance of a fluke or rostrum, parturition, and placental passage. Observations were initially conducted only during limited daytime hours; however, monitoring was changed to 24 h surveillance following the observation of parturition indicators.

This study was conducted using healthy beluga whales housed at the Port of Nagoya Public Aquarium. Animal procedures performed during the later phase of the study were approved by the Animal Care and Use Committee of Gifu University (No. 14094, 18 February 2015; No. 17186, 21 February 2018; No. 2020-264, 4 February 2021; No. AG-P-C-20240001, 4 April 2024). For pregnancies monitored before 2015, no formal institutional animal ethics approval process was available; however, all procedures were conducted as part of routine veterinary care and reproductive health management rather than for research purposes, thereby minimizing any additional stress or impact on the animals. Retrospective ethical approval was not available for these earlier records. Throughout the study period, we adhered to the Japanese Association of Zoos and Aquariums (JAZA) guidelines to ensure the ethical treatment and welfare of the animals. Informed owner consent was obtained from the facility responsible for the animals used in this study.

### 2.2. Rectal Temperature Measurement

Rectal temperature measurements were typically conducted between 09:00 and 10:00 as part of the routine reproductive monitoring. All measurements were performed by inserting a rectal probe (CTM-303, Terumo, Tokyo, Japan, and N543, Nikkiso-therm Co., Ltd., Tokyo, Japan) into the rectum. In four dams that delivered five live-born calves, voluntary cooperative behaviors were maintained until 2 days before parturition (DL-3), 10 days before parturition (DL-4), the day of parturition (DL-8 and DL-11), and 1 day before parturition (DL-6). Temperature assessments were conducted under voluntary cooperative behavior without restraint. To evaluate prepartum changes in rectal temperature, the mean rectal temperature during the latter half of the third trimester was used as the reference value for each pregnancy. Although preconception temperature data were available for all individuals, the latter half of the third trimester was selected because the primary objective was to assess temporal temperature changes within pregnancy as parturition approached, rather than to compare preconception and pregnancy values. This approach also helped minimize the influence of inter-individual variation.

### 2.3. Serum Progesterone Concentration Measurement

Blood samples were obtained from five adult female beluga whales during voluntary presentation or routine venipuncture as part of medical or management procedures. Sampling was conducted approximately once every 2 weeks, with adjustments made as appropriate based on medical or management considerations. Blood collection was performed as part of routine health monitoring, and the sampling interval varied slightly depending on the clinical condition and training status of each individual. Following collection, blood samples were centrifuged, and serum samples were stored at 4 °C prior to submission to the clinical laboratory. Samples were submitted for analysis on the day of collection or the following available business day. Samples were not frozen, and no freeze–thaw cycles occurred prior to progesterone measurement. Serum progesterone concentrations were measured using electrochemiluminescence immunoassay at the same clinical testing company throughout the study period (Nagoya Rinsho, Nagoya, Japan), minimizing inter-laboratory variation.

### 2.4. Ultrasonographic Measurements

Ultrasonography was performed for reproductive monitoring, including ovarian examinations and assessment of fetal viability through fetal heart rate detection. All examinations were conducted in conscious animals under voluntary husbandry behaviors. For ovarian examinations, whales were trained to float at the water surface with either the left or right lateral aspect of the body exposed, enabling transcutaneous scanning from the body wall. For fetal examinations, in addition to lateral presentations, whales were trained to assume a ventral-up position at the water surface, facilitating transabdominal ultrasonographic assessment. Ovarian follicles were identified in both sagittal (cranial–caudal) and transverse (dorsal–ventral) planes to monitor follicular development and confirm ovulation. Fetal examinations were performed according to methods described previously [1,4,5].

Examinations throughout gestation were only feasible in dams DL-5 and DL-9 because routine ultrasonographic monitoring had not been fully established during the earlier pregnancies. Examinations were conducted biweekly throughout gestation in dams DL-5 (newborn ID: DL-8 and DL-13) and DL-9 (newborn ID: DL-14). The fetal heart rate was recorded at each examination. To minimize handling time and reduce animal burden, the fetal heart rate was determined by counting cardiac beats over a 15 s interval and multiplying by four to obtain beats per minute (bpm). As fetal activity was occasionally associated with transient increases in fetal heart rate, measurements were obtained during periods of apparent fetal rest when possible to minimize movement-related variability. A 15 s counting interval was selected because prolonged maintenance of the required body position was frequently challenging despite long-term husbandry training, limiting the duration of continuous visualization of the fetal heart. When feasible, the frequency of ultrasonography was increased with impending parturition to enable closer monitoring of fetal viability. Ultrasonographic examinations were attempted two to three times per day during the expected peripartum period, although examinations were not consistently conducted because of animal behavior and husbandry conditions.

Cranial asymmetry was evaluated by measuring the distance from the intracranial midline structure (falx cerebri) to the left and right sides of the fetal cranium on transverse ultrasonographic images. No predefined quantitative threshold for abnormal asymmetry was applied. Different examiners were involved in DL-8 versus DL-13 and DL-14, with direct handover between examiners to maintain consistency as much as possible. Formal inter-observer repeatability was not assessed; however, ultrasonographic findings were evaluated repeatedly during serial examinations conducted throughout pregnancy. In the calf with suspected hydrocephalus (DL-13), asymmetry observed ultrasonographically was subsequently confirmed by gross postmortem examination, postmortem computed tomography, and necropsy findings, which demonstrated ventriculomegaly and corresponding cranial abnormalities.

### 2.5. Statistical Analysis

Data are presented as mean ± standard deviation (SD). Descriptive statistics, including mean and SD for rectal temperature and serum progesterone concentrations, were calculated using Microsoft Excel (Microsoft Corporation, Redmond, WA, USA). No inferential statistical analyses were performed because of the limited sample size, unequal sampling frequency among pregnancies, and heterogeneity of pregnancy outcomes. Therefore, this study should be interpreted as a descriptive case-series analysis rather than a hypothesis-testing study.

## 3. Results

### 3.1. Gestation Length

#### 3.1.1. Pregnancy Characteristics

The gestation length was 466 ± 8.4 days (range: 455–477 days). One abortion occurred on 13 December; copulatory behavior was not observed in this case, and the gestation length could not be determined. One preterm birth with hydrocephalus and additional indicators of developmental immaturity, including a persistent urachus, occurred on 17 April (gestation length: 422 days), which was 33–55 days shorter than those observed in successful pregnancies. A complete postmortem examination, including PMCT and necropsy, was performed on the hydrocephalic calf. No evidence of *Brucella* spp. or *Toxoplasma gondii* infection was identified, and no notable abnormalities were identified in the placenta. One stillbirth occurred on 7 March (gestation length: 480 days), which slightly exceeded the upper range of live births.

#### 3.1.2. Rectal Temperature Measurement

**Figure 1:**
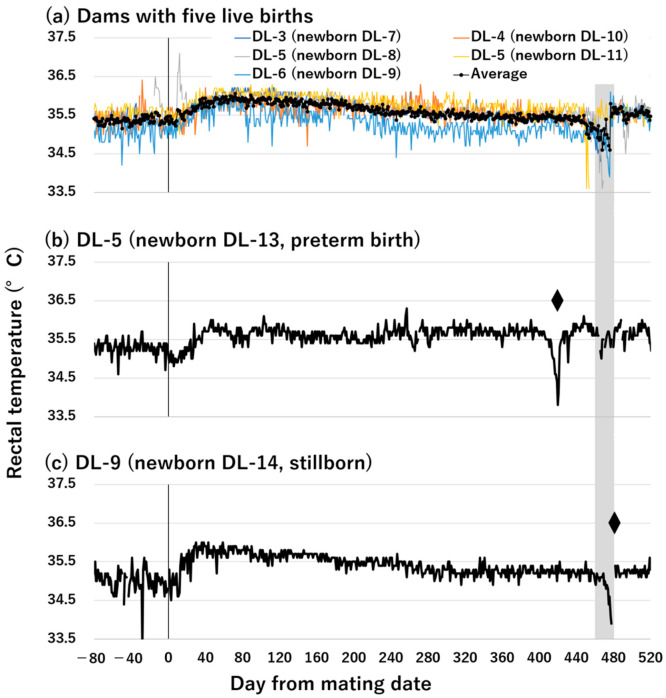
Changes in rectal temperature during pregnancy in beluga whales (*Delphinapterus leucas*). The maternal rectal temperature was measured daily; however, measurements were not possible on certain days immediately before parturition and during the early postpartum period until the maternal condition stabilized after the onset of nursing. Although rare, certain measurements were unavailable during gestation. (**a**) Rectal temperature profiles of four dams that produced five live-born calves. Colored lines represent individual pregnancies, and black circles indicate the mean rectal temperature of the five successful pregnancies. (**b**) Rectal temperature profile of dam DL-5 during the pregnancy resulting in preterm calf DL-13. (**c**) Rectal temperature profile of dam DL-9 during the pregnancy resulting in the stillbirth of newborn DL-14. The vertical black line indicates the estimated onset of pregnancy (Day 0). The gray shaded area represents the period of expected parturition based on the mean gestation length of successful pregnancies (Days 455–477). Black diamonds indicate the actual day of parturition in abnormal pregnancies.

The rectal temperatures of five dams with live births increased during early pregnancy, peaked in the first trimester, and subsequently declined gradually and remained relatively stable (Figure 1, Table 2). This parameter peaked within the first trimester and then declined to a relatively stable plateau throughout mid-gestation (Figure 1).

#### 3.1.3. Serum Progesterone Concentration Measurement

**Figure 2:**
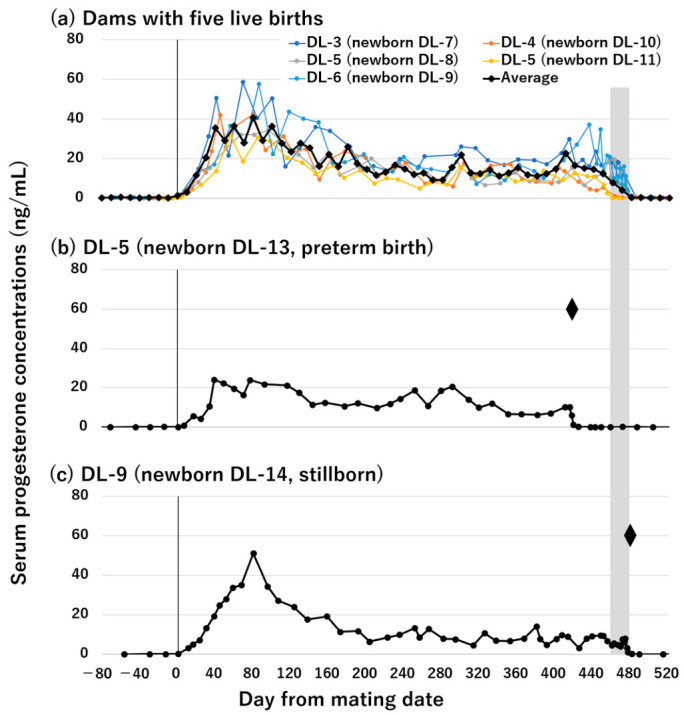
Changes in serum progesterone concentrations during pregnancy in beluga whales (*Delphinapterus leucas*). The maternal serum progesterone concentrations were measured once every 2 weeks. In certain cases, measurements were not possible on certain days immediately before parturition and during the early postpartum period until the maternal condition stabilized after the onset of nursing. Although rare, these measurements were unavailable during gestation. (**a**) Serum progesterone concentration profiles of four dams that produced five live-born calves. Colored lines represent individual pregnancies, and black circles indicate the mean serum progesterone concentration of the five successful pregnancies. (**b**) Serum progesterone concentration profile of dam DL-5 during the pregnancy resulting in preterm calf DL-13. (**c**) Serum progesterone concentration profile of dam DL-9 during the pregnancy resulting in the stillbirth of newborn DL-14. The vertical black line indicates the estimated onset of pregnancy (Day 0). The gray shaded area represents the period of expected parturition based on the mean gestation length of successful pregnancies (Days 455–477). Black diamonds indicate the actual day of parturition in abnormal pregnancies.

The serum progesterone concentrations in all the pregnant beluga whales remained elevated throughout gestation (Figure 2 and Table 3). During the first trimester, the serum progesterone concentrations exhibited considerable interindividual variability. Although considerable variability was observed among individuals, progesterone concentrations were maintained throughout mid and late gestation. Some adverse pregnancy outcomes were accompanied by lower progesterone concentrations than those generally observed in successful pregnancies.

#### 3.1.4. Ultrasonographic Measurements

Ultrasonographic measurements were performed on fetuses DL-8, DL-13, and DL-14. Their rates at 150–101, 100–51, and 50–0 days prepartum were 90.0 ± 8.49, 79.7 ± 23.33, and 54.0 ± 10.43 bpm for DL-8; 99.4 ± 5.55, 90.5 ± 3.54, and 81.0 ± 11.56 bpm for DL-13; and 89.0 ± 4.58, 70.0 ± 6.60, and 62.6 ± 9.90 bpm for DL-14, respectively. Cranial asymmetry was identified in DL-13 during late gestation, with the distance from the falx cerebri to the left parietal bone exceeding that to the right parietal bone. Similar findings were not observed in DL-8 or DL-14.

### 3.2. Periparturient Observations

#### 3.2.1. Periparturient Characteristics

**Figure 3:**
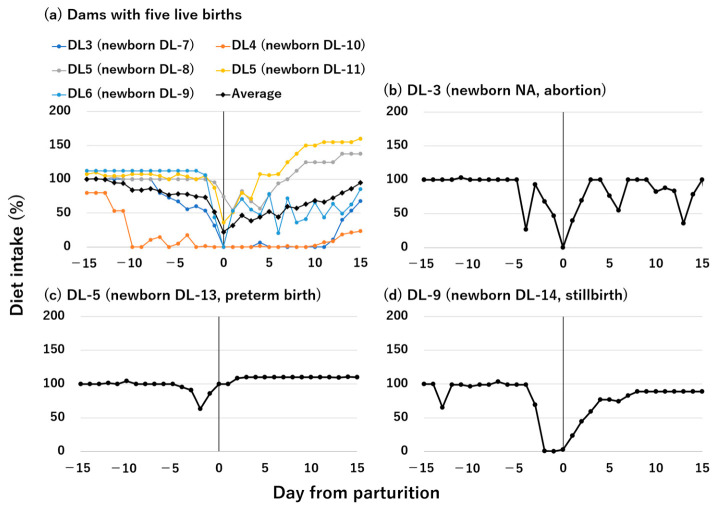
Changes in diet intake 15 days before and after parturition (Day 0) in captive beluga whale (*Delphinapterus leucas*) pregnancies. (**a**) Five live-born pregnancies (colored lines, individual pregnancies; black line, mean). (**b**) Abortion (DL-3 [newborn NA]). (**c**) Preterm calf (DL-5 [newborn DL-13]). (**d**) Stillbirth (DL-9 [newborn DL-14]). The vertical line indicates the day of parturition. NA, not applicable.

In four dams that delivered five live-born calves, milk leakage and membrane rupture were observed 2–10 and 0–1 days prepartum, respectively (Table 4). Vaginal discharge to fluke appearance, fluke appearance to parturition, and parturition to placental passage intervals were 0.7–6.8, 1.7–4.3, and 6.8–16.6 h, respectively (Table 1). Anorexia occurred gradually in two and acutely in three of the successful pregnancies (Figure 3a).

In dam DL-5, which delivered a preterm birth with hydrocephalus (DL-13), the interval from parturition to placental passage was prolonged to 20.8 h (Table 1). In dam DL-9, which delivered a stillbirth (DL-14), the interval from membrane rupture to fluke appearance was extended to 20.9 h, whereas the interval from parturition to placental passage was reduced to 1.5 h (Table 1). Additionally, minimal vaginal discharge, which may be associated with membrane rupture, was observed 3 days prepartum, following which liquid resembling sticky urine was continuously observed. All dams, except for DL-5, did not consume feed on the day of parturition, including dams that delivered surviving and dead fetuses (Figure 3a–d). The environmental and husbandry conditions specific to the single facility where this study was conducted may have influenced the observed physiological and behavioral patterns.

#### 3.2.2. Rectal Temperature Measurement

**Figure 4:**
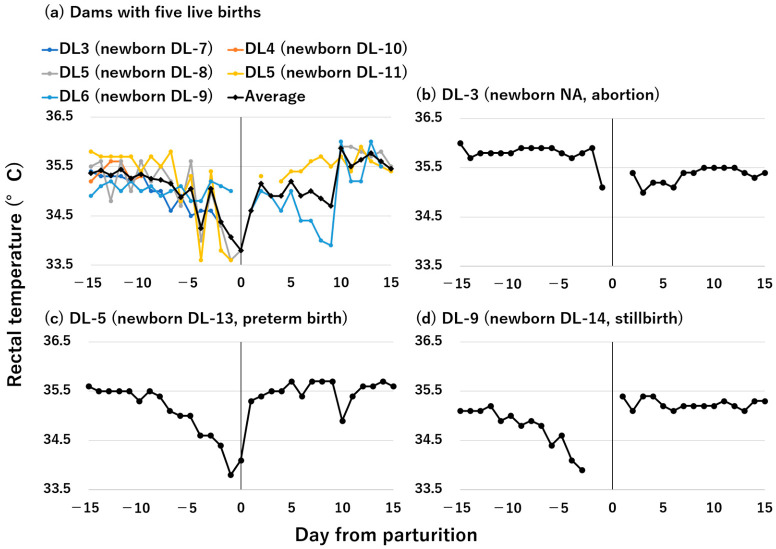
Changes in rectal temperature 15 days before and after parturition (Day 0) in captive beluga whale (*Delphinapterus leucas*) pregnancies. (**a**) Five live-born pregnancies (colored lines, individual pregnancies; black line, mean). (**b**) Abortion (DL-3 [newborn NA]). (**c**) Preterm calf (DL-5 [newborn DL-13]). (**d**) Stillbirth (DL-9 [newborn DL-14]). The vertical line indicates the day of parturition. NA, not applicable.

In two cases, the rectal temperature decreased by more than 1.0 °C from the baseline prepartum (Figure 4a). Among the dams that delivered dead newborns, DL-3 and DL-5 exhibited minimal differences in rectal temperatures. However, the rectal temperature of DL-9 progressively decreased beginning 11 days prepartum and reached more than 1.0 °C from the baseline 4 days before parturition (Figure 4b–d). No notable prepartum decline in rectal temperature was observed in DL-3, which resulted in abortion (Figure 4b).

Overall, the rectal temperatures of the dams that delivered live-born calves decreased 4 days before parturition, increased 3 days before parturition, and subsequently declined (Figure 4a). The rectal temperature decreased by −1.6 ± 0.5 °C on −1.3 ± 0.5 days in the dams that delivered live-born calves. The rectal temperature of DL-5, which delivered preterm calf DL-13, was 1.8 °C lower than that of the baseline temperature (Figure 4c). By contrast, the rectal temperature of DL-9, which delivered a stillbirth (DL-14), showed changes similar to those in the dams that delivered five live-born calves (Figure 4d).

#### 3.2.3. Serum Progesterone Concentration Measurement

**Figure 5:**
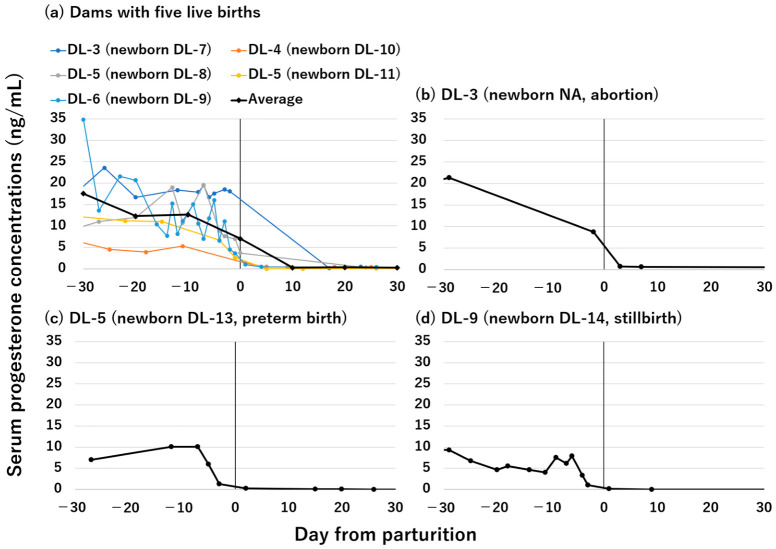
Changes in serum progesterone concentrations 15 days before and after parturition (Day 0) in captive beluga whale (*Delphinapterus leucas*) pregnancies. (**a**) Five live-born pregnancies (colored lines, individual pregnancies; black line, mean). (**b**) Abortion (DL-3 [newborn NA]). (**c**) Preterm calf (DL-5 [newborn DL-13]). (**d**) Stillbirth (DL-9 [newborn DL-14]). The vertical line indicates the day of parturition. NA, not applicable.

A decline in serum progesterone concentration was observed in all well-sampled dams before parturition. (Figure 5a). At 0–1 day prepartum, the serum progesterone concentrations ranged from 2.6 to 3.7 ng/mL (DL-5 [both for newborns DL-8 and DL-11] and DL-6) in all three well-sampled dams with live births. In DL-9, the serum progesterone concentrations decreased to 3.28 ng/mL 4 days prepartum and further declined to 1.07 ng/mL 3 days prepartum (Figure 5d). A decline in the serum progesterone concentrations was observed 2 days before parturition in the dams with live births; however, concentrations were maintained above 2.6 ng/mL until parturition (Figure 5a). By contrast, DL-5 (prepartum birth DL-13) and DL-9 (stillbirth DL-14) exhibited reduced serum progesterone concentrations (1.30 and 1.07 ng/mL, respectively) 3 days before parturition (Figure 5c,d).

#### 3.2.4. Ultrasonographic Measurements

Ultrasonographic evaluation demonstrated that, in DL-13 (dam ID: DL-5), the measured distance from the midline echo of the falx to the left temporal region was consistently greater than that to the right, particularly during the prepartum period. By contrast, no left–right asymmetry in biparietal width was detected in the live-born fetus DL-8 and stillbirth DL-14. DL-8 and DL-14 exhibited a progressive decline in fetal heart rate during the period preceding parturition. By contrast, DL-13 maintained relatively increased fetal heart rates during the final 50 days prepartum. The fetal heart rates of DL-13 and DL-14 were continuously monitored until 3 and 4 days prepartum, respectively. Owing to the decreased food intake and lethargy in dam DL-9, ultrasonography was not performed 4 days before parturition because the voluntary cooperative behavior required for the examination could not be maintained.

## 4. Discussion

The present study provides a long-term longitudinal evaluation of pregnancy and parturition in a managed population of beluga whales. Rectal temperature, as well as endocrine, behavioral, and ultrasonographic parameters, were integrated across a 25-year dataset. Copulatory activity and parturition were concentrated in spring, supporting a seasonal reproductive pattern in this managed beluga population. Previous studies have indicated that male calves experience significantly longer gestation periods than those of female calves (478 ± 8.6 vs. 457 ± 3.9 days) [14]. By contrast, here, among the normal parturitions at the PNPA, gestation length did not follow this previously reported sex-associated pattern. Owing to the limited sample size, particularly the presence of only one female calf, sex-associated trends should be interpreted with caution. Despite this discrepancy in sex-associated trends, the overall mean gestation length observed at PNPA (466 ± 8.4 days) closely resembled the previously reported value of 467 ± 5.4 days in the USA [14]. This concordance supports the typical biological consistency of gestation length across managed beluga populations while underscoring that deviations from this range may require closer clinical attention. However, published longitudinal datasets describing pregnancy and parturition in beluga whales remain limited [3,14], highlighting the importance of additional long-term facility-based reference datasets. Structured periparturient monitoring protocols have been proposed for bottlenose dolphins [2]; however, comparable evidence-based frameworks remain poorly defined for beluga whales. The present findings address this limitation by providing species-specific longitudinal data that inform multimodal monitoring strategies.

Vaginal discharge is a clinical indicator of impending parturition. However, visual assessment alone cannot reliably distinguish small amounts of mucus or blood from the true rupture of membranes. In DL-9, the dam of the stillbirth DL-14, discharge indicating mild membrane rupture was observed several days before delivery, although differentiation from urine was not consistently possible. Subsequently, changes in the abdominal contour suggested fetal repositioning toward parturition; however, ultrasonographic confirmation was not feasible because the dam had already developed anorexia. Notably, the interval between suspected membrane rupture and delivery appeared prolonged in this case. In human obstetrics, prolonged latency (>24 h) between membrane rupture and parturition at term is associated with increased maternal and neonatal morbidity [20]; however, this has not been reported in cetaceans. Direct extrapolation from human obstetrics to cetaceans should be approached cautiously, and the prolonged prepartum interval observed in this case may require further investigation.

In the dams with live births, the rectal temperature notably declined immediately before parturition. In one killer whale, the maternal body temperature decreased by approximately 0.4 °C 2 days prepartum and by 0.7 °C 1 day prepartum at one facility in Japan [10]. In bottlenose dolphins, birth normally occurs 24 h after a decrease in temperature of 1 °C [2]. Comparable declines within 12–24 h prepartum have also been observed in four bottlenose dolphins at one facility in Japan [11]. These findings suggest that rectal temperature monitoring may provide valuable clinical information regarding impending parturition in managed cetaceans. In the present study, rectal temperature decreased by ≥1 °C within several days before parturition in both successful and unsuccessful cases, indicating that this decrease may be associated with impending parturition. However, hypothermia may also develop secondary to systemic disease; therefore, a decline in rectal temperature should be interpreted in combination with other clinical findings [21,22,23]. Maternal body temperature does not consistently decrease prepartum; however, its decline is indicative of impeding parturition. Failure to deliver following this decrease should necessitate a thorough evaluation of both the maternal and fetal status.

The serum progesterone concentrations in the successful pregnancies showed a broadly consistent temporal pattern and were maintained at approximately 2–4 ng/mL even at term despite a prepartum decline. However, their utility was constrained by sampling difficulties in individuals with reduced food intake. By contrast, substantially reduced concentrations, including values approaching postpartum levels before parturition in one stillbirth case (DL-9), were observed in unsuccessful pregnancies. Reduced progesterone concentrations were typically observed in pregnancies with adverse outcomes, including preterm birth and stillbirth. However, the limited number of abnormal pregnancies in the present study precludes the establishment of clinically useful threshold values. These observations demonstrate that progesterone monitoring alone may have limited utility in predicting the exact timing of parturition; however, an excessive decline in serum progesterone concentrations before delivery may indicate abnormalities in the fetal condition or labor progression. Of note, a definitive progesterone threshold below which pregnancy can no longer be maintained was not established in the present study because of the limited number of cases.

The ultrasonographic assessment of fetal heart rate provides valuable information on fetal development prepartum, and serial examinations may facilitate the detection of developmental deviations prepartum, as previously reported in bottlenose dolphins [5]. Similar decreases in fetal heart rate have been reported in equine fetuses during late gestation [24]. In horses, these changes indicate maturation of the fetal autonomic nervous system and the development of cardiac regulatory mechanisms. However, its feasibility is limited by maternal conditions; in certain cases, anorexia and poor cooperation prevent the confirmation of fetal heart rate and the timing of fetal demise, highlighting a practical limitation in large marine mammals. Ultrasonography also enables the noninvasive detection of structural abnormalities suggestive of hydrocephalus. In the present study, the fetus affected by hydrocephalus (DL-13) showed persistent cranial asymmetry and relatively increased fetal heart rates during late gestation compared to those of the live-born and stillborn fetuses. However, because this observation was based on a single case, it should be considered hypothesis-generating. Although conclusions cannot be based on a single case, these findings indicate that serial ultrasonographic evaluation may facilitate the identification of developmental abnormalities before parturition. Therefore, ultrasonography is a valuable tool for monitoring fetal development in beluga whales; however, its diagnostic utility is influenced by practical and biological constraints.

The present findings underscore the value of integrating physiological, behavioral, endocrine, and ultrasonographic observations in managed beluga populations. Rectal temperature monitoring may provide a practical and noninvasive adjunct tool for anticipating parturition. Progesterone dynamics provide a reliable framework for staging pregnancy and may facilitate clinical assessment during late gestation, although they do not completely characterize the fetal health status. Changes in food intake may also serve as adjunctive indicators of impending parturition [5,6]. In all successful pregnancies, appetite decreased before parturition, although the timing and severity varied among individuals. Although progesterone concentrations also declined during late gestation, their relationship with appetite changes could not be evaluated directly in this retrospective study. However, the degree of prepartum appetite suppression varied among individuals, suggesting that this parameter should be interpreted with caution as a clinical monitoring parameter. The onset and severity of anorexia varied considerably among individuals, indicating that appetite alone is unlikely to provide a reliable predictor of the timing of parturition.

The availability of a long-term dataset covering multiple pregnancies within a single facility enabled the identification of consistent monitoring trajectories that may not be evident in short-term or cross-sectional studies. Ultrasonographic evaluation, particularly the assessment of cranial symmetry and fetal heart rate trends, provides critical information regarding structural and functional development. No single physiological, endocrine, behavioral, or ultrasonographic parameter consistently distinguished successful and unsuccessful pregnancies. However, deviations across multiple monitoring parameters were more frequently observed in pregnancies with adverse outcomes. Overall, these parameters appear to be most informative when interpreted collectively.

This study was limited by the small sample size and presence of only a single case each of preterm birth and stillbirth. Therefore, statistical comparisons were not feasible, and interpretations of abnormal findings remained primarily descriptive. Additional limitations include the use of data from a single facility, changes in equipment and husbandry practices over the 25-year study period, non-standardized monitoring intervals among pregnancies, and incomplete ultrasonographic coverage across all cases. Furthermore, facility-specific environmental and husbandry conditions may have influenced the observed physiological and behavioral patterns. Future studies with large sample sizes are required to establish clinically applicable thresholds for endocrine and ultrasonographic parameters. Collectively, this study provides preliminary evidence that the interpretation of multiple monitoring parameters may facilitate the clinical assessment and management of beluga whale pregnancies.

## 5. Conclusions

This study provides a long-term longitudinal description of pregnancy and parturition in a managed population of beluga whales and establishes a facility-specific reference pattern for prepartum monitoring. Multimodal monitoring proved valuable, as deviations from expected endocrine, behavioral, and ultrasonographic trajectories were more consistently associated with adverse pregnancy outcomes than with abnormalities in any single parameter. The rectal temperature decreased 1.3 ± 0.5 days before parturition, and serum progesterone concentrations typically followed a consistent gestational profile and were maintained at approximately 2–4 ng/mL until parturition in pregnancies resulting in live births. Ultrasonographic assessment of fetal heart rate and cranial morphology also provided valuable information regarding fetal development. These findings suggest that integrated multimodal monitoring may have practical value for reproductive management of beluga populations. Large multi-institutional datasets, standardized reproductive monitoring protocols, and collaborative longitudinal registries will be important in optimizing predictive thresholds and improving clinical decision-making.

## Figures and Tables

**Table 1:** Pregnancy details of dam beluga whales (*Delphinapterus leucas*).

Dam ID	Date of Birth	Pregnancy Check	Mating Date	Delivery Date	Pregnancy Period (Days)	Periparturitional Behavior (h)			Newborn ID	Sex	Status
						Stage 1	Stage 2	Stage 3			
DL-3	1989–1994 ^1^	NA	NA	13 December 2002	NA	NA	NA	NA	NA †	Male	Abortion
		P4, PPBO	6 April 2003	17 July 2004	468	1.4	4.3	6.8	DL-7	Male	Normal
DL-4	1998 ^1^	P4, PPBO	27 April 2009	1 August 2010	461	6.1	4.3	10	DL-10	Male	Normal
DL-5	1998 ^1^	P4, PPBO	9 April 2006	22 July 2007	469	0.7	1.7	10.3	DL-8	Male	Normal
		P4, PPBO	5 May 2011	2 August 2012	455	0.7	2.4	16.6	DL-11	Male	Normal
		P4, PPBO, US	17 April 2022	11 June 2023	420	3.3	1.3	20.8	DL-13 †	Female	Preterm birth Growth restriction Hydrocephalus
DL-6	1994 ^1^	P4, PPBO	4 April 2006	25 July 2007	477	6.8	2.3	7.6	DL-9	Female	Normal
DL-9	25 July 2007	P4, PPBO, US	7 March 2023	29 June 2024	480	20.9	4.6	1.5	DL-14 †	Male	Stillbirth

^1^ The expected date of birth was estimated on the basis of their body lengths [19]. † Indicates a fetus or calf that did not survive. The boundaries were defined based on gestational developmental stages reported previously [14]. Stage 1: Labor indicators with vaginal discharge, milk discharge, decreased appetite, contractions, decreased basal temperature, and increased intermammary distance; Stage 2: Starts with the appearance of flukes and finishes with the birth of the calf; and Stage 3: Complete expulsion of the placenta. DL-5 (newborn DL-13) had a longer third trimester than dams that delivered normal newborns. DL-9 (DL-14) had a longer first trimester and shorter third trimester than dams that delivered normal newborns. P4, serum progesterone concentration; PPBO, periparturitional behavior observation; US, ultrasonography; NA, not applicable.

**Table 2:** Rectal temperatures of dam beluga whales (*Delphinapterus leucas*).

Dam ID	(Newborn ID)	Rectal Temperature (°C) (Mean ± SD)							
			Days After Mating						
		Before Mating Date	First Trimester		Second Trimester		Third Trimester		
		−80 to −1	0 to 78	79 to 156	157 to 235	236 to 313	314 to 397	398 to 11 Days Before Parturition	Last 10 Days to Parturition
DL-3	(newborn DL-7)	35.4 ± 0.1	35.8 ± 0.3	36.0 ± 0.1	35.8 ± 0.1	35.6 ± 0.1	35.5 ± 0.1	35.4 ± 0.1	34.8 ± 0.3
DL-4	(newborn DL-10)	35.4 ± 0.2	35.7 ± 0.3	35.8 ± 0.2	35.7 ± 0.2	35.7 ± 0.2	35.5 ± 0.2	35.4 ± 0.2	35.3 *
DL-5	(newborn DL-8)	35.5 ± 0.3	35.8 ± 0.3	36.0 ± 0.1	35.8 ± 0.1	35.6 ± 0.1	35.5 ± 0.1	35.5 ± 0.2	34.8 ± 0.7
	(newborn DL-11)	35.6 ± 0.1	35.9 ± 0.3	36.0 ± 0.1	35.9 ± 0.1	35.8 ± 0.2	35.7 ± 0.2	35.7 ± 0.1	34.9 ± 0.9
	(newborn DL-13 †, preterm birth)	35.3 ± 0.2	35.5 ± 0.3	35.7 ± 0.2	35.6 ± 0.1	35.7 ± 0.2	35.7 ± 0.1	35.6 ± 0.1	34.8 ± 0.5
DL-6	(newborn DL-9)	35.1 ± 0.2	35.5 ± 0.3	35.5 ± 0.2	35.4 ± 0.2	35.1 ± 0.2	35.1 ± 0.2	35.1 ± 0.2	34.5 ± 0.4
DL-9	(newborn DL-14 †, stillbirth)	35.0 ± 0.3	35.6 ± 0.4	35.7 ± 0.1	35.5 ± 0.1	35.3 ± 0.2	35.2 ± 0.1	35.2 ± 0.1	34.6 ± 0.4

Mating date was set as Day 0. * Rectal temperature was measured only 10 days before parturition. SD, standard deviation. † Indicates a fetus or calf that did not survive.

**Table 3:** Serum progesterone concentrations (mean ± SD (*n*)) in dam beluga whales (*Delphinapterus leucas*).

Dam ID	(Newborn ID)	First Trimester		Second Trimester		Third Trimester		
		Days 0–78	Days 79–156	Days 157–235	Days 236–313	Days 314–397	Day 398 to 11 Days Before Parturition	Last 10 Days Before Parturition
DL-3	(newborn DL-7)	29.8 ± 21.6 (6)	33.5 ± 13.4 (5)	20.8 ± 7.9 (6)	21.2 ± 4.2 (4)	19.5 ± 3.0 (6)	20.5 ± 4.7 (8)	18.1 ± 0.9 (6)
DL-4	(newborn DL-10)	18.4 ± 14.5 (9)	26.0 ± 10.8 (6)	17.9 ± 5.1 (5)	11.6 ± 5.8 (6)	11.7 ± 4.1 (6)	8.5 ± 4.9 (6)	N/A
DL-5	(newborn DL-8)	14.0 ± 12.6 (6)	26.3 ± 7.7 (5)	17.0 ± 3.9 (6)	12.2 ± 4.0 (5)	8.9 ± 2.4 (5)	11.8 ± 4.2 (7)	9.4 ± 6.0 (5)
	(newborn DL-11)	12.3 ± 11.9 (6)	22.1 ± 7.6 (5)	11.3 ± 3.3 (6)	9.1 ± 4.2 (5)	11.1 ± 2.1 (5)	11.0 ± 1.5 (4)	4.7 ± 2.9 (2)
	(newborn DL-13 †, preterm birth)	12.7 ± 9.6 (10)	16.8 ± 4.8 (5)	11.7 ± 1.8 (5)	16.4 ± 4.0 (5)	8.0 ± 2.4 (6)	NA	6.9 ± 4.2 (4)
DL-6	(newborn DL-9)	19.6 ± 12.9 (5)	40.4 ± 12.6 (5)	17.4 ± 4.0 (6)	14.4 ± 5.3 (5)	12.4 ± 3.0 (5)	18.8 ± 9.5 (13)	9.6 ± 4.4 (9)
DL-9	(newborn DL-14 †, stillbirth)	16.9 ± 13.0 (10)	30.8 ± 12.8 (5)	11.2 ± 4.4 (6)	9.1 ± 3.4 (6)	8.4 ± 3.1 (7)	7.0 ± 2.3 (13)	5.2 ± 2.9 (5)

SD, standard deviation; units, ng/mL; NA, not applicable. † Indicates a fetus or calf that did not survive.

**Table 4:** Observed indicators of parturition in dam beluga whales (*Delphinapterus leucas*).

Dam ID	(Newborn ID)	Observed Indicators of Parturition (Days Prior to Parturition)			
		Arching and Crouching	Milk Leaking	Rupture of Membrane	Day of 24 h Observation Started
		>5 Times/h			
Mean ± SD of dams that delivered live-born calves		8.2 ± 2.2	4.3 ± 3.9	0.6 ± 0.5	3.4 ± 2.6
DL-3	(newborn NA †, abortion)	NA	NA	NA	NA
	(newborn DL-7)	9	2	1	4
DL-4	(newborn DL-10)	8	NA	1	2
DL-5	(newborn DL-8)	8	3	0	0
	(newborn DL-11)	5	2	0	4
	(newborn DL-13 †, preterm birth)	NA	NA	NA	1
DL-6	(newborn DL-9)	11	10	1	7
DL-9	(newborn DL-14 †, stillbirth)	6	4	3	7

SD, standard deviation; NA, not applicable. † Indicates a fetus or calf that did not survive.

## Data Availability

The data presented in this study are available upon reasonable request from the corresponding author.
